# Basophil activation test in cancer patient blood evaluating potential hypersensitivity to an anti‐tumor IgE therapeutic candidate

**DOI:** 10.1111/all.14245

**Published:** 2020-03-10

**Authors:** Heather J. Bax, Atousa Khiabany, Chara Stavraka, Giulia Pellizzari, Charleen Chan Wah Hak, Alexandra Robinson, Kristina M. Ilieva, Natalie Woodman, Cristina Naceur‐Lombardelli, Cheryl Gillett, Sarah Pinder, Hannah J. Gould, Christopher J. Corrigan, Stephen J. Till, Sidath Katugampola, Claire Barton, Anna Winship, Sharmistha Ghosh, Ana Montes, Debra H. Josephs, James F. Spicer, Sophia N. Karagiannis

**Affiliations:** ^1^ St. John’s Institute of Dermatology School of Basic & Medical Biosciences King’s College London London UK; ^2^ School of Cancer & Pharmaceutical Sciences King’s College London Guy’s Hospital London UK; ^3^ Departments of Medical Oncology and Clinical Oncology Guy’s and St Thomas’ NHS Foundation Trust London UK; ^4^ Breast Cancer Now Research Unit School of Cancer & Pharmaceutical Sciences Guy’s Cancer Centre King’s College London London UK; ^5^ King’s Health Partners Cancer Biobank School of Cancer & Pharmaceutical Sciences King’s College London London UK; ^6^ Randall Centre for Cell and Molecular Biophysics School of Basic and Medical Biosciences King's College London London UK; ^7^ Asthma UK Centre Allergic Mechanisms in Asthma King's College London London UK; ^8^ Department of Respiratory Medicine and Allergy and School of Immunology and Microbial Sciences King's College London London UK; ^9^ Centre for Drug Development Cancer Research UK London UK; ^10^ Barton Oncology Ltd Eastcote UK

**Keywords:** basophils, BAT, IgE, MOv18, ovarian cancer

To the Editor,

Monoclonal anti‐tumor IgG antibodies are used widely to treat malignancies. Studies in the field of AllergoOncology, focusing on the interactions between IgE, allergy, and cancer, point to biological characteristics of IgE that may engender potent anti‐tumor functions.^1^ These include superior affinity of IgE for cognate Fc receptors and the presence in tumors of effector cell populations (eg, macrophages and mast cells) known to exert anti‐tumor activities when activated by IgE.^2^, ^3^ Following promising preclinical findings,^2^, ^4^ MOv18 IgE, specific for the tumor‐associated antigen folate receptor alpha (FRα), overexpressed in ovarian and basal breast cancers and other solid tumors,^5^ is the first anti‐cancer IgE antibody studied in a first‐in‐class, first‐in‐human clinical trial (ClinicalTrials.gov Identifier: NCT02546921).

One of the potential concerns associated with application of IgE as a therapy in the clinic relates to the perceived risk of IgE‐mediated anaphylaxis. Safety evaluation of such a novel agent mandated the development of bespoke methods to monitor potential hypersensitivity reactions following intravenous infusion and ideally also to help in predicting such a reaction when selecting patients for treatment. Over the past 15 years, the basophil activation test (BAT) has been developed and widely employed to study and predict type 1 hypersensitivity reactions to food, venom, and drugs in the allergy field.^6^, ^7^ Thus far, its use in the context of cancer is limited to a small number of studies for the detection of allergic reactions to chemotherapeutic agents.^8^ Basophil activation in the context of tumor‐associated immunomodulation and in often heavily treated patients has not been well‐studied.

Employing the BAT in whole blood of 42 ovarian cancer patients with diverse treatment histories and tumor histologies, we examined the propensity of human basophils to be activated by anti‐cancer IgE ex vivo. We first identified circulating basophils (CCR3^high^SSC^low^; gating strategy in Figure S1A) from patients with cancer. Basophils were activated (up‐regulation of CD63 expression) ex vivo by IgE‐ and non‐IgE‐mediated triggers (anti‐FcεRI, anti‐IgE, and fMLP, Figure 1A, Figure S1B). Consistent with previously reported findings in allergic cohorts,^6^ levels of basophil activation varied among individuals*.* We detected no basophil activation following addition of the hapten‐specific NIP (4‐hydroxy‐3‐iodo‐5‐nitrophenylacetic acid) IgE alone or its multivalent antigen (NIP‐BSA) alone. However, we detected basophil activation by exogenous stimulation of the hapten‐specific NIP IgE in combination with multimeric NIP‐BSA (Figure 1A). This suggested that IgE could recognize unoccupied cell surface FcεRI on basophils ex vivo and basophils could be activated by exogenous FcεRI receptor engagement and formation of cross‐linking immune complexes.

**Figure 1:**
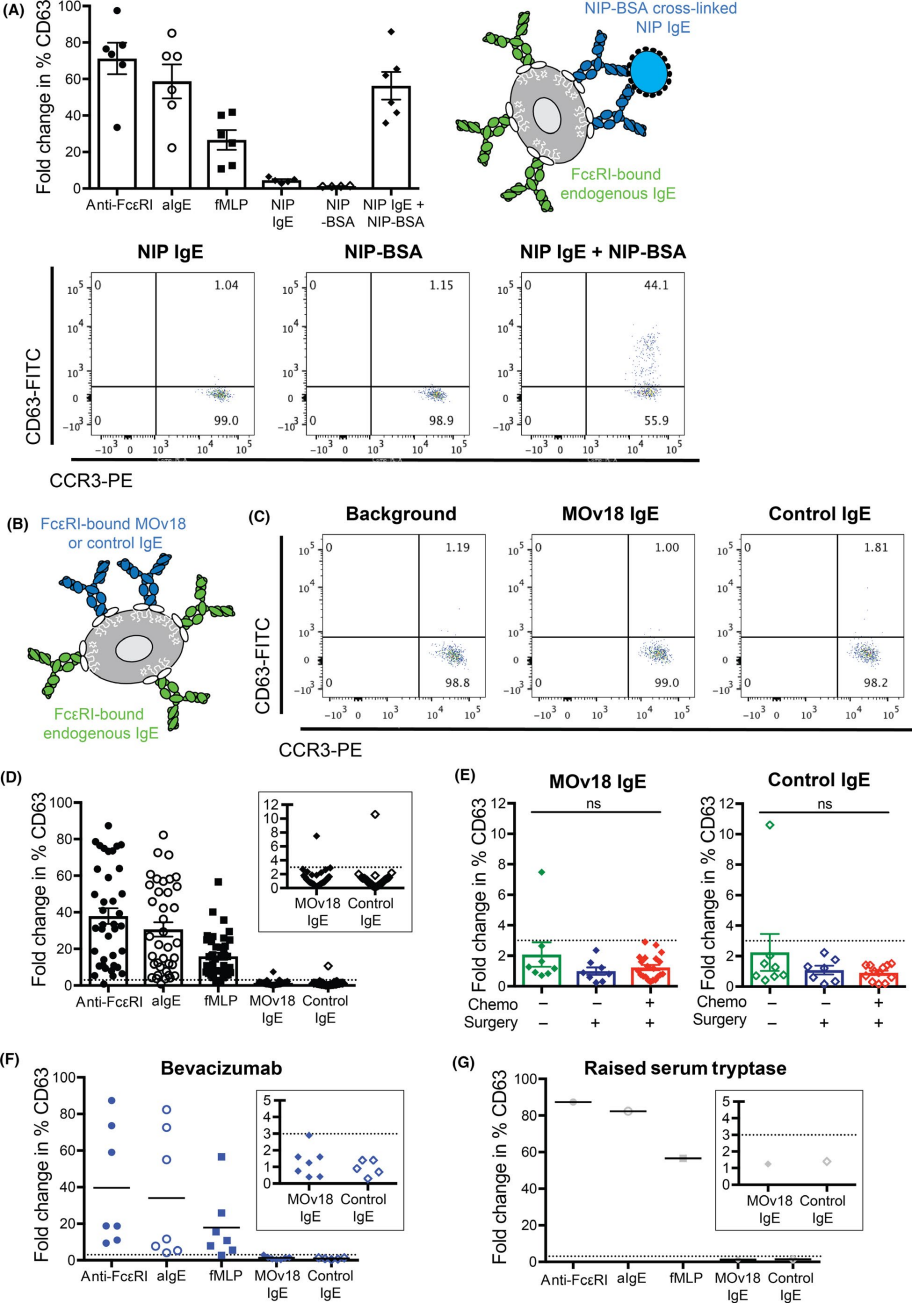
Anti‐cancer IgE does not trigger basophil activation in 98% of cancer patient blood samples studied. Basophil activation (fold change in % CD63 expression) was evaluated following stimulation with anti‐FcεRI antibody, anti‐IgE antibody, and fMLP (positive controls) and cross‐linking of NIP IgE by multimeric NIP‐BSA (A). No basophil activation (<3.0 fold change of % CD63‐positive basophils, dotted cutoff line) was triggered by MOv18 or control IgE in all but one specimen, despite activation by positive controls (B‐D), and irrespective of previous standard treatments received (E, F), nor when measured in the blood of a patient with already raised serum tryptase (G)

We then examined whether stimulation with the anti‐cancer mouse/human chimeric IgE antibody (MOv18) could trigger ex vivo basophil activation (Figure 1B, C). As expected in this cohort (n = 42), stimulation with anti‐FcεRI, anti‐IgE, and fMLP (positive controls) triggered CD63 up‐regulation. In all but one patient sample, no basophil activation was measured following incubation of ovarian cancer patient blood with MOv18 IgE or control non‐FRα‐reactive IgE in the absence of any additional exogenous cross‐linking stimulus (mean fold change in %CD63: 1.4 for MOv18 IgE, 1.3 for control IgE; 7.5 and 10.6, respectively, in the positive responder) (Figure 1D). Activation, or lack thereof, was irrespective of different patient tumor histologies and treatment histories, that is, (a) treatment‐naïve patients (n = 7), (b) following primary debulking surgery (n = 8), (c) following surgery and chemotherapy (n = 21), or (d) following treatment with bevacizumab (n = 7) (Figure 1E, F). Neither MOv18 IgE nor control non‐FRα‐reactive IgE triggered basophil activation in the blood of a patient with already raised serum tryptase, a marker which could indicate mastocytosis (although this clinical information was not available) and may have potentially predisposed this individual to an increased risk of hypersensitivity to IgE stimulation, including to MOv18 IgE (Figure 1G).

Since MOv18 IgE recognizes the tumor‐associated antigen, FRα, it is possible that FRα shed from cancer cells in tissues and anti‐FRα autoantibodies (autoAbs), if present in patient circulation, could form immune complexes with MOv18 IgE. This may result in FcεRI cross‐linking and basophil activation (Figure 2A). No CD63 up‐regulation on basophils was measured following ex vivo stimulation with either MOv18 IgE or control IgE in any sample from patients with known tumor FRα expression status, as determined by immunohistochemistry (Figure 2B, C, Table S1). Anti‐FRα IgE autoAbs were not detectable in patient serum (Table S1). Although serum FRα and anti‐FRα IgG autoAbs were measurable in 44% and 21% of patients, respectively (Figure 2D, F, Table S1), basophils in 41 of 42 matched unfractionated blood samples were not activated by incubation with MOv18 or control IgE (Figure 2E, G). MOv18 IgE combined with monovalent recombinant FRα did not trigger activation (Figure S1C). Moreover, no MOv18 IgE‐mediated activation was measured in those 9% of patients with both measurable serum FRα and IgG autoAbs against FRα, or in the blood from 2 of the 3 patients who additionally had FRα‐positive tumor (Figure 2H, Table S1). Basophil activation by MOv18 IgE was observed in only one patient. In this patient's blood sample, we measured circulating FRα but no anti‐FRα autoAbs. The patient's tumor FRα expression status was unknown, and serum tryptase levels were not elevated (7 ng/mL; Table S1). In the same patient, CD63 up‐regulation was also triggered by the control non‐FRα‐reactive IgE. Together, these suggested that basophil activation in this specimen may involve a non‐FRα‐specific mechanism, potentially through a humoral response directed toward the antibody's structural components. The prevalence of such a propensity to activate basophils in ovarian cancer and other patient cohorts and its potential clinical significance require further in‐depth investigations. Such studies may consider the possible cross‐linking by autoAbs, such as those recognizing alpha‐gal (galactose‐α‐1,3‐galactose) previously associated with hypersensitivity to cetuximab, an anti‐EGFR IgG antibody,^9^ or by anti‐drug antibodies (ADAs) that may develop following MOv18 IgE treatment.

**Figure 2:**
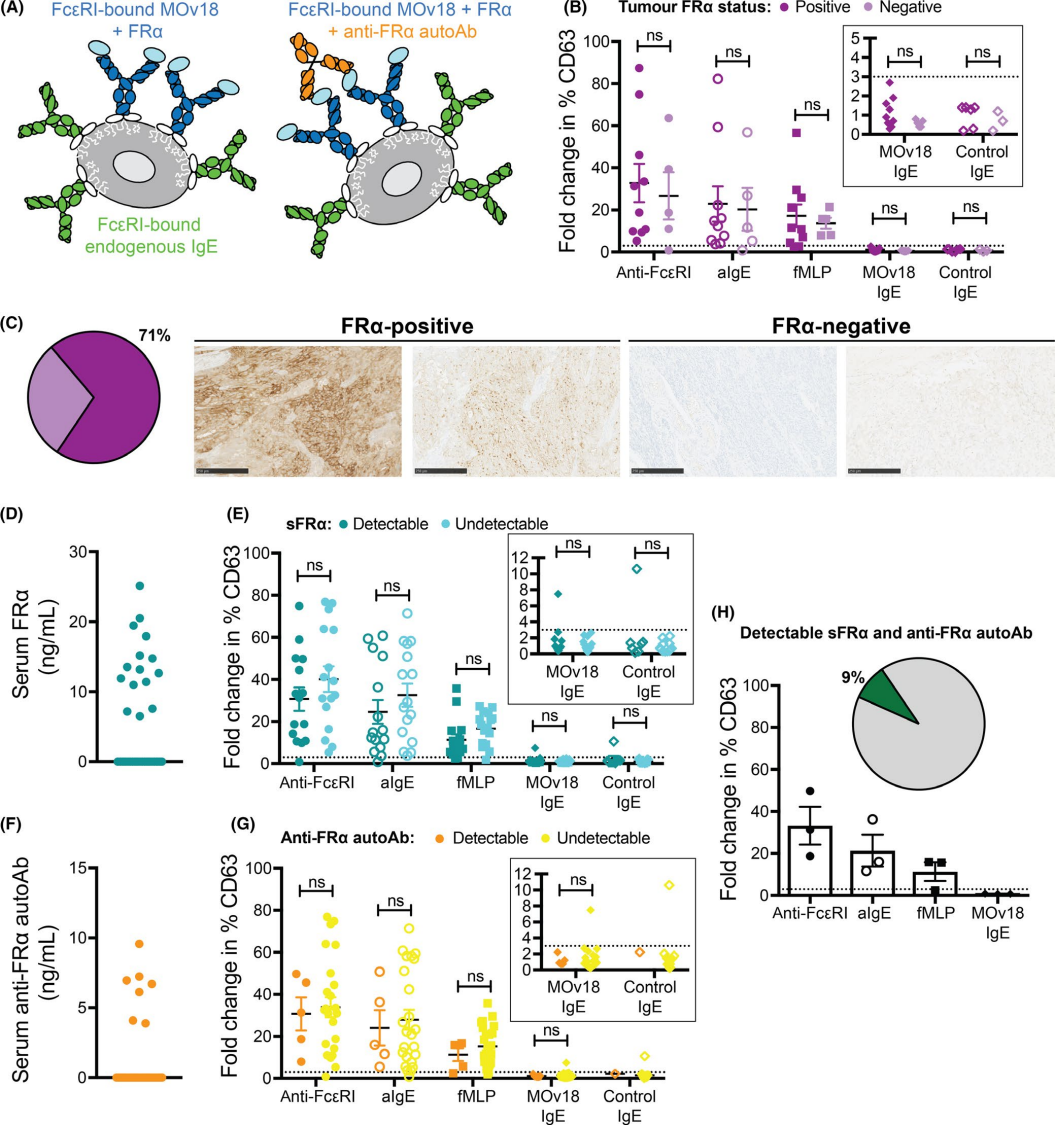
FRα‐positivity in blood or tumor does not influence basophil activation by anti‐cancer IgE. Circulating FRα and anti‐FRα autoantibodies may form immune complexes with MOv18 IgE, triggering basophil activation (A). No basophil activation was measured following MOv18 IgE stimulation in blood from the 71% of patients with FRα‐positive tumor (B) (representative FRα‐stained paraffin‐embedded tumor, C). Despite detectable FRα or anti‐FRα IgG autoantibodies in a proportion of patients, MOv18 IgE triggered basophil activation in one blood sample (D‐G). In the 9% of patients with both FRα and anti‐FRα IgG autoantibodies, no basophil activation by MOv18 IgE or control IgE was observed (H)

In conclusion, the basophil activation test showed no reactivity with MOv18 or control IgE in 41 of 42 ovarian cancer patients’ samples. Combined with measurements of other clinical and biological parameters, application of BAT to the clinical study of a first‐in‐class IgE in cancer patients (ClinicalTrials.gov Identifier: NCT02546921) may allow correlations with clinical observations, to help monitor and potentially predict patient safety.

## CONFLICTS OF INTEREST

SNK and JFS are founders and shareholders of IGEM Therapeutics Ltd., and HJB is now employed through a fund provided by IGEM Therapeutics Ltd. CB is a freelance pharmaceutical physician/medical advisor with Barton Oncology Ltd and in addition to work with Cancer Research UK Centre for Drug Development has undertaken consultancy work with many companies including in the last ~5 years, Astex Therapeutics Ltd, BerGen Bio A/S, Cancer Targeting Systems Inc, CellCentric Ltd, Certara LP, EngMab AG, Inbiomotion SL, Innate Pharma SA, Macrophage Pharma Ltd, MorphoSys AG, Mosaic Biomedicals SL, Norgine Pharmaceuticals Ltd, Ono Pharma UK Ltd, Orion Clinical Services Ltd, PIQUR Therapeutics AG, PTEN Research Foundation, SFL Services GmBH, Shionogi Ltd, T3 Pharmaceuticals AG, UCB Biopharma SPRL, and the Wellcome Trust Ltd. CB is on the advisory board for SFL Services GmBH and owns shares in GlaxoSmithKline. All other authors have declared no conflict of interest.

## FUNDING INFORMATION

The authors acknowledge support from Cancer Research UK (C30122/A11527; C30122/A15774); the Academy of Medical Sciences; CRUK//NIHR in England/DoH for Scotland, Wales and Northern Ireland Experimental Cancer Medicine Centre (C10355/A15587), the Inman Charity, the Medical Research Council (MR/L023091/1), the Cancer Research UK King's Health Partners Centre at King's College London, and Breast Cancer Now (147) working in partnership with Walk the Walk. The research was supported by the National Institute for Health Research (NIHR) Biomedical Research Centre (BRC) based at Guy's and St Thomas' NHS Foundation Trust and King's College London (IS‐BRC‐1215‐20006). The authors are solely responsible for study design, data collection, analysis, decision to publish, and preparation of the manuscript. The views expressed are those of the author(s) and not necessarily those of the NHS, the NIHR, or the Department of Health.

## ETHICAL APPROVAL

This study has been reviewed and approved by Guy's Research Ethics Committee (Reference 09/H0804/45).

## Supporting information

supinfo
