# Supplementary material for: Basophil activation test in cancer patient blood evaluating potential hypersensitivity to an anti‐tumor IgE therapeutic candidate
Source: Allergy. 2020 Mar 10;75(8):10.1111/all.14245. doi: 10.1111/all.14245 (PMC7581190; doi:10.1111/all.14245)
Supplement: Supplementary file 1 — supinfo [file ALL-75-na-s001.docx]

**Supplementary Methods**

***Ovarian cancer patient study***

Women with ovarian cancer were enrolled into the study by written informed consent. Peripheral venous blood samples were drawn into BD Vacutainer™ Hemogard Closure Plastic K2-EDTA Tubes (BD). Serum samples were prepared by drawing blood into SST Clot Activator and Polymer Gel Hemogard Closure Blood Tubes (BD), followed by centrifugation of clotted blood at 2500RPM for 15 minutes at 4°C, careful pipetting of serum and storage at -80°C. Demographic characteristics, including tumor histology and prior treatment history, were obtained from clinical databases, anonymized and analyzed in conjunction with clinical samples.

***Basophil Activation Test (BAT)***

The basophil activation test (BAT) was performed within 4 hours of blood collection using the Flow2 CAST^®^ kit (Bühlmann) as per instructions, except that incubation time with stimuli was optimized from the recommended 10 minutes to 30 minutes. Briefly, unfractionated whole blood was incubated with stimulation buffer (Bühlmann), and different stimuli: anti-FcεRI (Bühlmann), anti-IgE antibody (Dako) or fMLP (Bühlmann), or anti-FRα antibody, MOv18 IgE or control non-FRα-reactive IgE antibodies (at 3.5 μg/ml, prepared in-house). In the case of hapten-specific anti-NIP IgE, cross-linking with NIP-BSA (at 20 μg/ml, 5 NIP to BSA ratio, in-house) was included. Monovalent recombinant FRα (R&D Systems) was added, at indicated concentrations, to some MOv18 IgE stimulations. All conditions were then stained with anti-CCR3-PE and anti-CD63-FITC staining cocktail (Bühlmann) and incubated at 37°C for 30 minutes in a 5% CO_2_ incubator. For all preparations, red blood cell lysis was then performed with diluted lysis buffer (Bühlmann) for 10 minutes at room temperature, followed by centrifugation and resuspension of cell pellets with acquisition buffer (Bühlmann). Flow cytometric evaluations were performed with a FACSCanto™ II using FACSDiva software (BD). Basophil activation was expressed as the fold change in % CD63-positive CCR3-PE^high^SSC^low^ basophils over the background control (stimulation buffer and staining antibody cocktail alone) for each sample^1^. The % CD63 expression (without fold change calculation) is also shown in Figure S1B. All data analyses were performed, and representative plots prepared using FlowJo™ software (FlowJo LLC).

***Tumor FRα expression status by immunohistochemistry (IHC)***

For a subset of patients, tumor sections from primary debulking surgery were evaluated for FRα expression status. Novocastra^TM^ Liquid mouse anti-human FRα primary antibody (Leica) was applied to formalin-fixed, paraffin-embedded tumor sections (from primary debulking surgery) for 32 minutes at room temperature at 1/500 dilution, followed by detection with Ultra Universal 3,3'-diaminobenzidine (DAB) detection kit (Ventana Medical Systems Inc.) and then Haematoxlyin II applied for 8 minutes. This protocol was performed using the BenchMark ULTRA automated immunohistochemistry/in situ hybridisation (IHC/ISH) slide staining system (Ventana Medical Systems Inc.), with an extended cell conditioning 2 (CC2) solution antigen retrieval.

***Circulating FRα and anti-FRα autoantibody ELISAs***

ELISAs were performed as previously described^2^. Circulating FRα (*e.g.* shed from tumor tissues) or anti-FRα autoantibodies in ovarian patient serum samples were evaluated by first coating 96-well MaxiSORP™ plates (Nunc) with 100 μl/well of 2 μg/ml monoclonal mouse anti-human FRα IgG1 antibody (clone 548908) or 1 μg/ml recombinant FRα, respectively (both R&D Systems and diluted in 0.2M carbonate-bicarbonate buffer, Pierce). Following incubation at 4°C, overnight, plates were blocked with 250 μl/well SuperBlock^TM^ (Perbio Science Ltd.) for 2 hours at room temperature and then washed 4 times with 250 μl/well PBS-0.05% Tween® 20 solution (Severn Biotech and Sigma, respectively). Serum samples were diluted to 20% (or to 50% for IgE autoantibodies) in a 50:50 solution of SuperBlock^TM^ and PBS-0.05% Tween® 20. Standard curves of recombinant FRα (R&D Systems), or anti-FRα human IgG or IgE monoclonal antibody (prepared in house) were diluted in SuperBlock^TM^-PBS-0.05% Tween® 20, supplemented with 20% human serum albumin (type AB male, Sigma). Samples and standards were added 50 μl/well, in triplicate, and incubated for 2 hours at room temperature, followed by 4 washes. FRα was detected by 50 μl/well addition of biotinylated polyclonal goat anti-human FRα IgG1 antibody (R&D Systems, diluted to 25 ng/ml in SuperBlock^TM^-PBS-0.05% Tween® 20) for 2 hours at room temperature, 4 further washes, and 50 μl/well addition of streptavidin-peroxidase conjugate (Pierce, diluted 1/22000 in SuperBlock^TM^-PBS-0.05% Tween® 20) for 30 minutes at room temperature. Anti-FRα IgG autoantibodies were detected by 50 μl/well addition of HRP-conjugated polyclonal goat anti-human Fcγ-specific F(ab’)_2_ fragment (Jackson Immuno Research, diluted 1/500 in SuperBlock^TM^-PBS-0.05% Tween® 20) for 45 minutes at room temperature. Anti-FRα IgE autoantibodies were detected by 50 μl/well addition of HRP-conjugated polyclonal goat anti-human IgE antibody (Sigma, diluted 1/500 in SuperBlock^TM^-PBS-0.05% Tween® 20) for 2 hours at room temperature. Plates were then washed 5 times and developed by 50 μl/well addition of OPD (Sigma) diluted to 0.5 mg/ml in stable peroxidase substrate buffer (Pierce) for 5-10 minutes, at room temperature, in darkness, followed by 50 μl/well 1M HCl solution (Sigma). Using a Fluostar Omega microplate reader (BMG LABTECH), FRα and anti-FRα IgG autoantibodies were measured using an absorbance 492nm, with a correction wavelength of 650nm. Standard curves were fitted using a 4-point variable curve-fitting program using a minimum of 6 points (MARS software, BMG LABTECH). The lower limit of quantification (LLOQ) was 6.25 ng/ml, 3.125 ng/ml, and 5 ng/ml for FRα, anti-FRα IgG, and anti-FRα IgE, respectively. Values below LLOQ are reported as 0 ng/ml.

***Statistical Analyses***

All statistical analyses were performed in GraphPad Prism (GraphPad Software, Inc.). Datasets were compared by t-test or one-way ANOVA with Kruskal-Wallis multiple comparisons. P values were represented as follows: *=P<0.05, **=P<0.01, ***=P<0.001, ****=P<0.0001. Error bars represent Standard Error of Mean (SEM).

***References***

1. Fernandez TD, Ariza A, Palomares F, et al. Hypersensitivity to fluoroquinolones: The expression of basophil activation markers depends on the clinical entity and the culprit fluoroquinolone. *Medicine (Baltimore).* 2016;95(23):e3679.

2. Rudman SM, Josephs DH, Cambrook H, et al. Harnessing engineered antibodies of the IgE class to combat malignancy: initial assessment of FcvarepsilonRI-mediated basophil activation by a tumour-specific IgE antibody to evaluate the risk of type I hypersensitivity. *Clin Exp Allergy.* 2011;41(10):1400-1413.

**
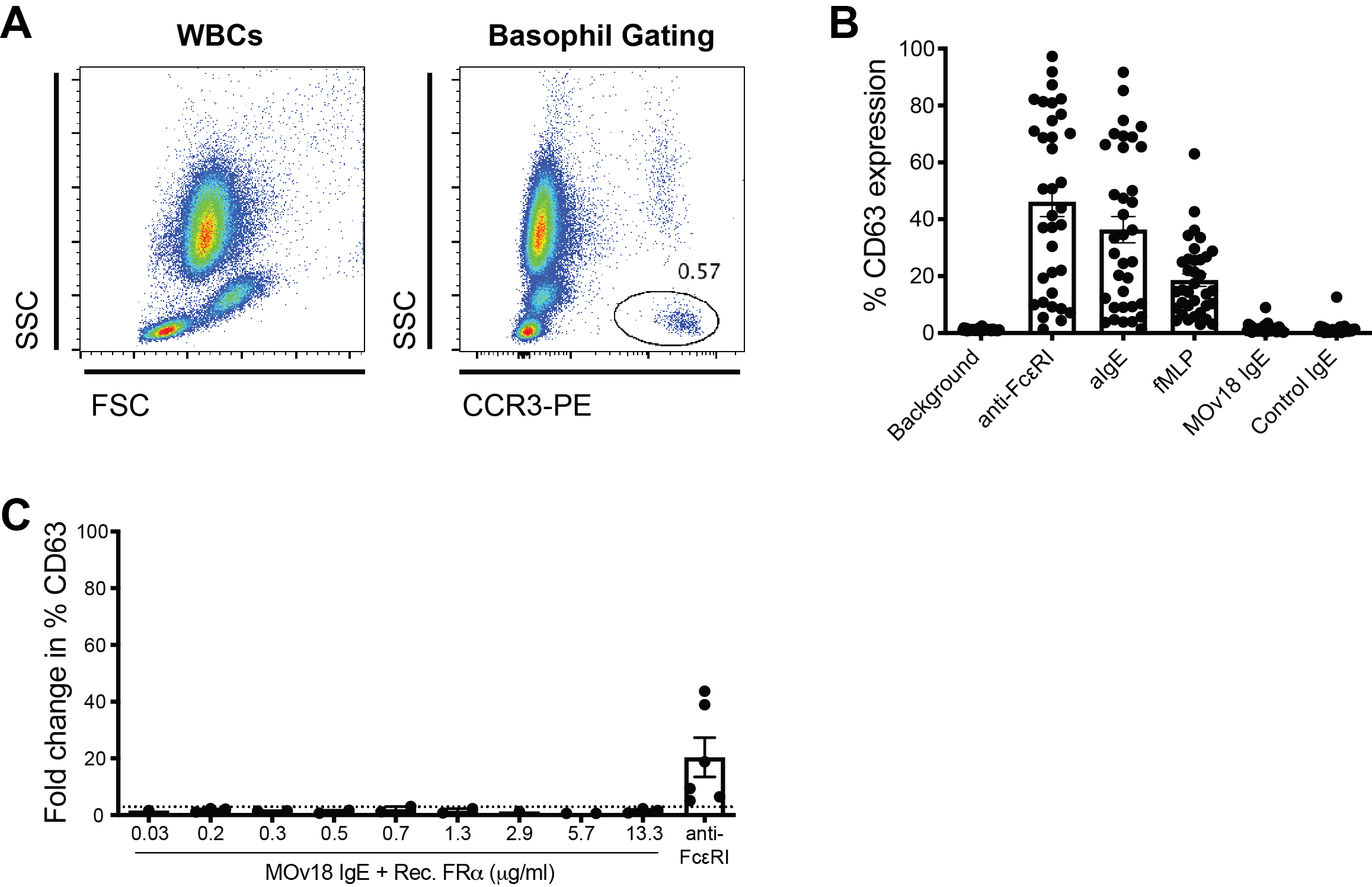
**

**Figure S1– (A) Basophil Activation Test (BAT) gating strategy.** As per instructions for the Flow2 CAST^®^ kit (Bühlmann), basophils in unfractionated whole blood samples were gated as CCR3^high^SSC^low^. Up-regulation of CD63 on the surface of basophils was monitored as a marker of *ex vivo* basophil activation; **(B) Basophil Activation Test (BAT) % CD63 expression without fold change calculation**; **(C) MOv18 IgE combined with recombinant FRα does not trigger basophil activation in patient blood.** No basophil CD63 up-regulation was measured following *ex vivo* stimulation with MOv18 IgE, plus monovalent recombinant FRα at concentrations up to 13.3 μg/ml, 500-fold higher than those measured physiologically in ovarian cancer patient circulation (highest FRα 25.13 ng/ml measured in our cohort (Figure 2D, Table S1)).

**Table S1 – Ovarian cancer patient characteristics.**

|  | **FRα status** | | | | **BAT Fold change in %CD63** | |
| --- | --- | --- | --- | --- | --- | --- |
|  | **Tumor FRα expression** | **sFRα (ng/ml)** | **Anti-FRα IgG autoAb (ng/ml)** | **Anti-FRα IgE autoAb (ng/ml)** | **MOv18 IgE** | **Control non-FRα-reactive IgE** |
| 1 | Positive | 7.19 | 0.00 | - | 1.9 | - |
| 2 | Negative | 7.58 | 6.71 | - | 0.8 | - |
| 3 | - | 11.42 | 0.00 | - | 1.0 | - |
| 4 | - | 25.13 | 0.00 | - | 2.9 | - |
| 5 | - | 0.00 | 0.00 | - | - | - |
| 6 | - | 0.00 | 0.00 | - | 1.2 | - |
| 7 | - | 0.00 | 3.90 | - | 2.2 | - |
| 8^†^ | Positive | 0.00 | 9.57 | - | - | - |
| 9 | - | 19.47 | 0.00 | 0.00 | 7.5 | 10.6 |
| 10 | - | 11.90 | 4.10 | 0.00 | 0.9 | - |
| 11 | Positive | 13.19 | 0.00 | 0.00 | 0.6 | - |
| 12 | Negative | 0.00 | 0.00 | 0.00 | 0.6 | - |
| 13 | Positive | 20.52 | 6.13 | 0.00 | 0.7 | - |
| 14^†^ | Positive | 0.00 | 7.23 | - | - | - |
| 15 | Negative | 0.00 | 0.00 | 0.00 | 0.4 | 0.7 |
| 16^†^ | - | 0.00 | 0.00 | 0.00 | - | - |
| 17 | - | 0.00 | 6.96 | 0.00 | 1.2 | 2.2 |
| 18 | - | 0.00 | 0.00 | 0.00 | 2.4 | 1.8 |
| 19 | Negative | 6.51 | 0.00 | 0.00 | 0.4 | 0.2 |
| 20 | Positive | 0.00 | 0.00 | 0.00 | 0.3 | 0.2 |
| 21 | - | 0.00 | 0.00 | 0.00 | 0.2 | 0.2 |
| 22 | - | 0.00 | 0.00 | 0.00 | 1.5 | 1.3 |
| 23 | Negative | 0.00 | 0.00 | 0.00 | 0.7 | 1.2 |
| 24 | - | 0.00 | 0.00 | 0.00 | 1.3 | 2.0 |
| 25 | - | 0.00 | 0.00 | 0.00 | 2.6 | 0.8 |
| 26 | - | 0.00 | 0.00 | 0.00 | 0.8 | 0.4 |
| 27 | - | 0.00 | 0.00 | 0.00 | 0.6 | 0.3 |
| 28 | - | 0.00 | 0.00 | 0.00 | 1.2 | 0.7 |
| 29 | Positive | 14.75 | 0.00 | 0.00 | 0.9 | 1.3 |
| 30 | - | - | - | - | 0.8 | 0.6 |
| 31 | - | 12.67 | 0.00 | - | 1.6 | 1.5 |
| 32 | - | 15.16 | 0.00 | - | 0.7 | 0.7 |
| 33 | Positive | 13.54 | 0.00 | - | - | - |
| 34 | - | - | - | - | 0.8 | 1.2 |
| 35 | - | - | - | - | 0.3 | 0.6 |
| 36 | Positive | - | - | - | 1.6 | 1.4 |
| 37 | Positive | 17.95 | 0.00 | - | 0.4 | 0.3 |
| 38 | Positive | 10.99 | 0.00 | - | 2.7 | 1.4 |
| 39 | - | - | - | - | 1.8 | 1.0 |
| 40 | - | - | - | - | 1.6 | 0.9 |
| 41 | - | - | - | - | 1.9 | 1.5 |
| 42^‡^ | Positive | - | - | - | 1.3 | 1.4 |

^†^’Non-responder’ patients, ^‡^Patient with elevated serum tryptase (33 ng/ml; ULN = 14 ng/ml). –Not tested.
